# Dispersed DNA variants underlie hearing loss in South Florida’s minority population

**DOI:** 10.1186/s40246-023-00556-7

**Published:** 2023-11-24

**Authors:** LéShon Peart, Joanna Gonzalez, Dayna Morel Swols, Duygu Duman, Turcin Saridogan, Memoona Ramzan, Mohammad Faraz Zafeer, Xue Zhong Liu, Adrien A. Eshraghi, Michael E. Hoffer, Simon I. Angeli, Guney Bademci, Susan Blanton, Carson Smith, Fred F. Telischi, Mustafa Tekin

**Affiliations:** 1 Dr. John T. Macdonald Foundation Department of Human Genetics, University of Miami Miller School of Medicine, Miami, FL USA; 2 Department of Audiology, Faculty of Health Sciences, Ankara University, Ankara, Turkey; 3 Hussman Institute for Human Genomics, Miller School of Medicine, University of Miami, Miami, FL USA; 4 Department of Otolaryngology, Miller School of Medicine, University of Miami, Miami, FL USA

**Keywords:** Genetic, Gene variants, Multicultural, Minority, Racial and ethnic, Sensorineural hearing loss

## Abstract

**Background:**

We analyzed the genetic causes of sensorineural hearing loss in racial and ethnic minorities of South Florida by reviewing demographic, phenotypic, and genetic data on 136 patients presenting to the Hereditary Hearing Loss Clinic at the University of Miami. In our retrospective chart review, of these patients, half self-identified as Hispanic, and the self-identified racial distribution was 115 (86%) White, 15 (11%) Black, and 6 (4%) Asian. Our analysis helps to reduce the gap in understanding the prevalence, impact, and genetic factors related to hearing loss among diverse populations.

**Results:**

The causative gene variant or variants were identified in 54 (40%) patients, with no significant difference in the molecular diagnostic rate between Hispanics and Non-Hispanics. However, the total solve rate based on race was 40%, 47%, and 17% in Whites, Blacks, and Asians, respectively. In Non-Hispanic Whites, 16 different variants were identified in 13 genes, with *GJB2* (32%), *MYO7A* (11%), and *SLC26A4* (11%) being the most frequently implicated genes. In White Hispanics, 34 variants were identified in 20 genes, with *GJB2* (22%), *MYO7A* (7%), and *STRC-CATSPER2* (7%) being the most common. In the Non-Hispanic Black cohort, the gene distribution was evenly dispersed, with 11 variants occurring in 7 genes, and no variant was identified in 3 Hispanic Black probands. For the Asian cohort, only one gene variant was found out of 6 patients.

**Conclusion:**

This study demonstrates that the diagnostic rate of genetic studies in hearing loss varies according to race in South Florida, with more heterogeneity in racial and ethnic minorities. Further studies to delineate deafness gene variants in underrepresented populations, such as African Americans/Blacks from Hispanic groups, are much needed to reduce racial and ethnic disparities in genetic diagnoses.

**Supplementary Information:**

The online version contains supplementary material available at 10.1186/s40246-023-00556-7.

## Background

Hearing loss (HL) is a global health concern affecting approximately 1.5 billion people with a projected increase to 2.5 billion by 2050 [1, 2]. It affects ~ 1 newborn in every 500 births in developed countries [3, 4]. In at least 50% of these individuals, it is assessed to be of genetic etiology [5]. Genetic HL can be syndromic (30% of inherited HL) or non-syndromic (NS; 70% of inherited HL). Autosomal recessive (AR) inheritance is the most common form of inheritance, accounting for up to 80% of individuals [4, 6, 7]. Autosomal dominant (AD) inheritance is seen in approximately 20%, with the remaining 5% belonging to X-linked (XL) and mitochondrial inheritance forms [8].

Previous studies have shown that the etiological diagnostic rate after genetic testing of genes and variants varies broadly between ethnic and racial groups [9, 10]. For instance, *GJB2* variants have been reported as the most common cause in people of European and Asian ancestry [11, 12]. On the other hand, *GJB2* variants are rare in Black populations [13–15]. Florida is the third most populous state of the U.S. and two-thirds of the South Floridian population is Hispanic/Latino and half of whom are foreign-born [16]. Despite the 14.9% prevalence of hearing impairment in the state of Florida, detailed studies of deafness genes in a South Florida population have not been reported [17–19]. Caribbean Hispanics (Cuban, Puerto Rican, and Dominican) make up the majority of this population and the remaining is largely from Central and South America other than Mexico [20]. The Black population of South Florida is also quite diverse with one-third of Blacks being foreign-born (including Haiti and English-speaking Caribbean countries such as the Bahamas, Jamaica, and Trinidad). Other immigrant groups include people from the Middle East, and Central, South, and East Asia. In this study, we present demographic and phenotypic data in relation to causal gene variants in a diverse population of South Florida.

## Results

### Demographic data

Among the patients, 68 were female and 68 were male, with both groups ranging in age from 3 to 77 years old at the time of their last visit. The study population was ethnically diverse having an even divide, 68 (50%) Hispanic and 68 (50%) Non-Hispanic. The racial diversity of the population included 115 (86%) White, 15 (11%) Black, and 6 (4%) Asian. The self-reported ancestry of patients was highly diverse (Table 1). The Non-Hispanic Black population had ancestral origins from the U.S. and the Caribbean islands including Jamaica, Haiti, Bahamas, and Dominica. The Hispanic White cohort had many countries of origin ranging from Latin America, Europe, and the Caribbean. The ancestral origins of Non-Hispanic White cohort were from different regions of the U.S. with distant backgrounds from Europe. The Hispanic Black patients self-reported that their origins were from Cuba and Venezuela.

**Table 1:** Overall distribution of probands and identified gene variants

Race/ethnicity	Family origin	Gene	Inh	Variant DNA	Variant protein	Zyg
Asian/Non-Hispanic	Vietnam and India	*SIX1*	AD	c.533G > C	p.Arg178Thr	Het
Black/Non-Hispanic	African American/USA	*COL2A1*	AD	c.626G > A	p.Arg209Gln	Het
	Bahamas/Haiti/DR	*COL11A1*	AD	c.3816 + 1 G > A	N/A	Het
	Haiti/Denmark/Portugal/France	*EYA1*	AD	c.966 + 5 G > T	N/A	Het
	Dominica/Haiti + Bahamas	*OTOF*	AR	c.2122C > T	p.Arg708*	C. Het
				c.1966delC	p.Arg656Glyfs*10	
	African American	*OTOGL*	AR	c.2566_2569delAATT	p.Asn856Valfs*8	Het
				c.5992 + 5 G > A	N/A	
	African American/Jamaica	*RTN4IP1*	AR	c.308 G > A	p.Arg103His	C. Het
				c.890 A > G	p.Tyr297Cys	
	African American/Haiti	*MYO15A*	AR	c.8019delG	p.His2674Thrfs*64	Het
				c.8065delT	p.Trp2689Glyfs*49	
White/Hispanic	Colombia	*ATP6V1B1*	AR	c.1248 + 1G > C	N/A	Hom
	Puerto Rico	*CABP2*	AR	c.590 T > C	p.Ile197Thr	Hom
	Cuba	*COL11A1*	AD	c.5009_5013delGTTGG	p.Ser1670Ilefs*2	Het
	Venezuela/Nicaragua	*COL2A1*	AD	c.870 + 1G > A	N/A	Het
	Dominican Republic	*COL4A4*	AR	c.2219dupC	p.Val741Cysfs*47	Hom
	Cuba	*EYA1*	AD	c.1653 T > A	p.Tyr551*	Het
	Colombia	*GATA3*	AD	DEL_Chr10:460,302–11,345,840 (including *GATA3*)	N/A	Het
	Colombia/Jamaica/China	*GJB2*	AR	c.299_300delAT	p.His100Argfs*14	C. Het
				c.596 C > T	p. Ser199Phe	
	Brazil/Cuba	*GJB2*	AR	c.139 G > T	p.Glu47Ter	C. Het
				c.35delG	p.Gly12Valfs*2	
	Spain/Cuba/France	*GJB2*	AR	c.35delG	p.Gly12Valfs*2	Hom
	Cuba	*GJB2*	AR	c.35dupG	p.Val13Cfs*35	C. Het
				c.35delG	p.Gly12Valfs*2	
	Puerto Rico/Cuba	*GJB2*	AR	c.35delG	p.Gly12Valfs*2	Hom
	Cuba/Colombia	*GJB2*	AR	c.109 G > A	p.Val37Ile	C. Het
				c.35delG	p.Gly12Valfs*2	
	Colombia	*MT-RNR1*	Mit	m.1555A > G	N/A	Hmp
	Puerto Rico	*MYO15A*	AR	c.7226delC	p.Pro2409Glnfs*8	Hom
	Peru	*MYO7A*	AR	c.2263-1G > T	N/A	C. Het
				c.4920delC	p.Glu1842fs	
	Honduras	*MYO7A*	AR	c.73G > A	p.Gly25Arg	Hom
	Cuba/Spain	*OTOF*	AR	c.2485C > T	p.Gln829*	C. Het
			AR	c.2348delG	p.Gly783Alafs*17	
	Mexico	*OTOG*	AR	c.6559 C > T	p.Arg2187*	Het
				c.8047 + 3 G > T	N/A	
	Italy/Spain/Mexico	*PCDH15*	AR	c.4211 + 1G > T	N/A	C. Het
				c.3877C > T	p.Arg1293Trp	
	Cuba	*STRC* and *CATSPER2*	AR	15q15.3 Deletion	N/A	Hom
	Mexico/Sweden	*STRC* and *CATSPER2*	AR	15q15.3 Deletion	N/A	Hom
	Cuba/Spain/Europe AJ	*SUMF1*	AR	c.463 T > C	p.Ser155Pro	Het
				c.539 G > T	p.Trp180Leu	
	Italy/Greece/Cuba/Spain	*TBC1D24*	AD/AR	c.724 C > T	p.Arg242Cys	C. Het
				c.641 G > A	p.Arg214His	
	Cuba	*TMC1*	AR	c.236 + 1 G > A	N/A	C. Het
				c.1939 T > C	p.Ser647Pro	
	Cuba	*TMPRSS3*	AR	c.208delC	p.His70Thrfs*19	Hom
	Cuba/Argentina	*USH2A*	AR	c.7475C > T	p.Ser2492Leu	C. Het
				Deletion in Exon 70	N/A	
White/Non-Hispanic	Europe/Italy	*ACTG1*	AD	c.773C > T	p.Pro258Leu	Het
	Eastern Europe/Russia/AJ	*AIFM1*	XL	c.1412 G > A	p.Gly471Glu	Hem
	Europe	*CDH23*	AR	c.1515-12G > A	N/A	C. Het
				c.3598G > T	p.Asp1200Tyr	
	Colombia/White	*COL4A5*	XL	c.4976 + 3A > G	N/A	Hem
	Southern Europe/AJ	*GJB2*	AR	c.35delG	p.Gly12Valfs*2	C. Het
				DEL_Chr13:20,797,176–21105944	N/A	
	Ukraine	*GJB2*	AR	c.35delG	p.Gly12Valfs*2	Hom
	White	*GJB2*	AR	c.269 T > C	p.Leu90Pro	Het
				309 kb deletion GJB6-D43S1830	N/A	
	Scotland/Germany/Ireland Native American/American Eskimo	*GJB2*	AR	c.35delG	p.Gly12Valfs*2	Hom
	Europe	*GJB2*	AR	c.35delG	p.Gly12fs*2	Hom
	England/Wales/Scotland/Ireland	*GJB2*	AR	c.35delG	p.Gly12Valfs*2	Hom
	Poland/Hungary/Czech/AJ	*LARS2*	AR	c.180 G > C	p.Glu60Asp	Hom
	Palestine	*LRP2*	AR	c.11581 T > C	p. Cys3861Arg	Hom
	England/German/France	*MYO6*	AD/AR	c.2867 + 1 G > A	N/A	Het
	Russia	*MYO7A*	AR	c.5101 C > T	p.Arg1701*	Het
				c.849 + 1 G > A	N/A	Het
	Germany/England	*MYO7A*	AD	c.2164 G > C	p.Gly722Arg	Het
	Ireland/Netherlands	*SLC26A4*	AR	c.85G > C	p.Glu29Gln	Het
				c.1544 + 3_1544 + 6delGAGT	N/A	
	Germany/Ukraine/Italy/Mexico	*SLC26A4*	AR	c.165–1 G > A	N/A	Het
				c.707 T > C	p.Leu236Pro	
	Brazil	*WFS1*	AD	c.409_424dupGGCCGTCGCGAGGCTG	p.Val142Glyfs*110	Het
	England/Italy/Ireland/Scotland	*Mitochondrial genome*	Mit	m.8649_16084del17436	N/A	Htp. 15%

*Inh* Inheritance, *Zyg* Zygosity, *AD* Autosomal Dominant, *AR* Autosomal Recessive, *Mit* Mitochondrial, *Hem* Hemizygous, *Hom* Homozygous, *Het* Heterozygous, *C. Het* Compound Heterozygous, *Htp* Heteroplasmic, *Hmp* Homoplasmic, *AJ* Ashkenazi Jewish

### Diagnostic yield and gene distribution by ethnicity and race

**Fig. 1:**
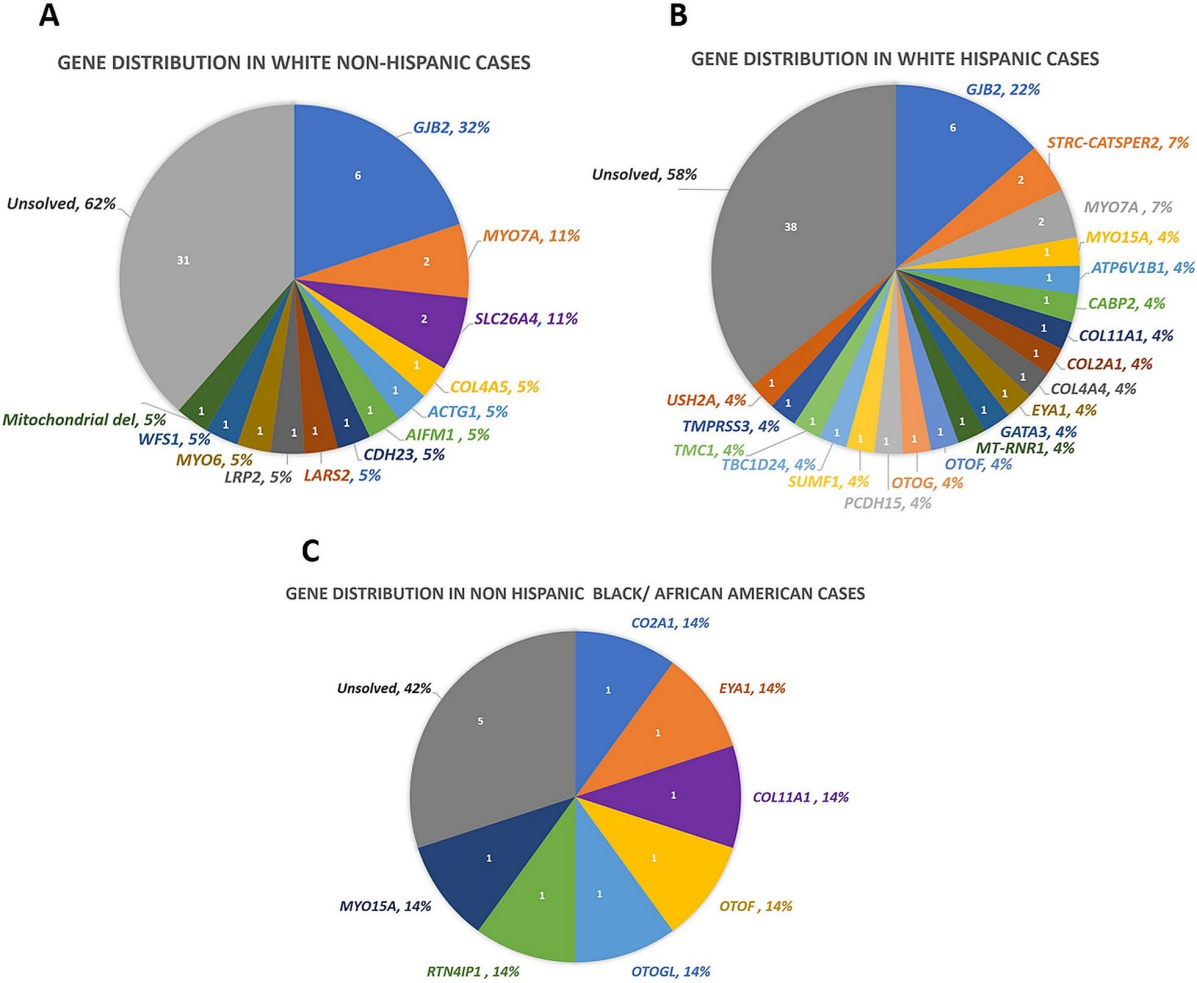
Causal Gene Distribution According to Racial and Ethnic Groups. **A** Exhibits the gene distribution in the solved White Non-Hispanic population. **B** Exhibits the gene distribution in the solved White Hispanic population. **C** Exhibits the gene distribution in the solved Non-Hispanic Black/African American population

**Fig. 2:**
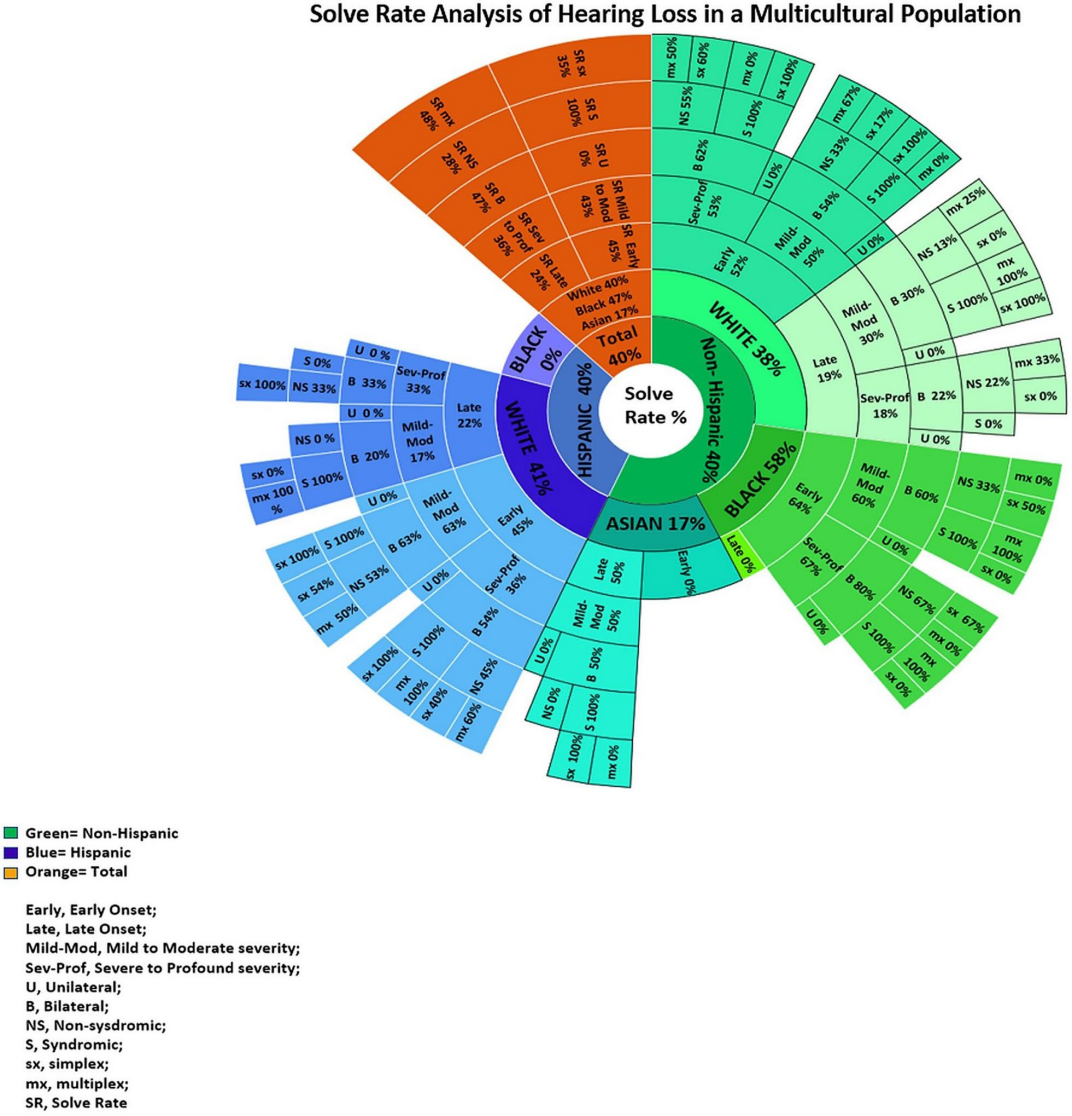
Solve Rate Analysis of Hearing Loss in a Multicultural Population. Solve Rate Analysis of ethnicity, race, phenotype, and family history of the Total, Non-Hispanic and Hispanic cohort. (The percentages are representative of the solve rate. i.e. Asian cohort has a 17% SR, of these if late onset HL the SR increases to 50%, when mild to moderate severity HL also present the SR remains at 50%, when bilateral HL also present the SR also remains at 50%, when the case is also syndromic the SR increases to 100% and remains at a 100% SR if no family history)

There was no significant difference in the solve rate (SR) based on ethnicity, with both Non-Hispanic and Hispanic groups having a SR of 40% (Figs. 1 and 2). Based on race, total SR was 40%, 47%, and 17% in Whites, African American/Blacks, and Asians, respectively (Figs. 1 and 2).

We solved (including those with possibly solved) 54 cases (40%) with 66 variants in 33 genes. Table 1, Additional file 1: Table S1 and Fig. 1 show the distribution of the genes and variants identified in different ethnic and racial groups. The overall gene distribution of the studied population shows that approximately half of the solved cases have variants in the top three genes: *GJB2* (22%, n = 12)*, MYO7A* (7%, n = 4), and *SLC26A4* (4%, n = 2)*.* A total of 16 gene variants causing sensorineural HL (SNHL) in the White Non-Hispanic cohort were *GJB2* (32%), *MYO7A* (11%), *SLC26A4* (11%) and single families (5%) in 10 other genes (Fig. 1A). In comparison, the White Hispanic cohort had a total of 34 gene variants identified as the cause of SNHL. The gene distribution showed that the most common genes are *GJB2* (22%), *STRC-CATSPER2* (7%), and *MYO7A* (7%) (Fig. 1B). The remaining 17 genes were detected in single families*.* The Non-Hispanic Black cohort had a total of 7 genes and 11 variants all identified as the cause of SNHL (Fig. 1C). The gene distribution in the Non-Hispanic Black Cohort was evenly dispersed, all occurring at one gene per family for variants in 7 genes (14% each). For the Asian cohort one gene variant in *SIX1* in one SNHL proband out of 6 patients was diagnostic.

### Diagnostic yield of phenotypic subgroups

The diagnostic rate varied with phenotypic groupings such as the age of onset, severity, laterality, and family history (Fig. 2). The SR differed between the age of onset groups. The late-onset group had an SR of 24% (n = 8) compared to the SR of 45% (n = 46) in those having early onset HL. The severity of HL had a slight difference in SR. Mild to Moderate HL had a higher SR of 43% compared to Severe to Profound HL which had an SR at 36%. SNHL laterality drastically affected the diagnostic yield. Bilateral SNHL had a SR of 47% (n = 54) compared to a 0% (n = 0) SR if unilateral SNHL was present (Fig. 2).

Probands who had syndromic SNHL had an SR of 100% (n = 22) compared to the non-syndromic SNHL phenotype which had an SR of 28% (n = 32). Syndromic SNHL included Stickler (18%, n = 4), Usher (18%, n = 4), Alport (9%, n = 2), Baraitser-Winter (4.5%, n = 1), *AIFM1-*related (4.5%, n = 1), Branchiootorenal (BOR 1) (9%, n = 2), Hypoparathyroidism, Sensorineural deafness, and Renal dysplasia (HDR) (4.5%, n = 1), Perrault (4.5%, n = 1), Donnai Barrow (4.5%, n = 1), Kearns-Sayre (4.5%, n = 1), *RTN4IP1*-related (4.5%, n = 1), Branchiootic (BOR 3) (4.5%, n = 1), Pendred (4.5%, n = 1), and multiple sulfatase deficiency (4.5%, n = 1) syndromes (Additional file 1: Table S1).

The SNHL simplex and multiplex cases exhibited a moderate difference in SR. Simplex cases had a diagnostic yield of 35% (n = 31) in comparison to 48% (n = 23) in the multiplex cases. In cases with a family history of AR HL the SR was highest at (70%, n = 38) compared to other inheritance patterns, AD (22%, n = 12), mitochondrial (4%, n = 2), and XL (4%, n = 2) (Additional file 1: Table S1).

## Discussion

In this report, we present the genetic causes of SNHL in a diverse population from South Florida. Overall, our diagnostic yield is 40%, which is similar to that previously reported in the U.S. with mixed racial and ethnic backgrounds [9]. While there appears to be a difference based on race, with African Americans/Blacks having a slightly higher SR compared to Whites, we do not see a difference in the SR between Hispanic and Non-Hispanic groups.

As with previous studies, we found variants in *GJB2* as the most common cause of SNHL, explaining 10% of our probands. While this percentage is smaller compared to that of previous reports from the U.S., it should be noted that our cohort contained patients with syndromic, unilateral, and mild-moderate HL, which are not commonly caused by *GJB2* variants [21]. When we included only probands with non-syndromic, early onset, severe to profound, and bilateral SNHL, *GJB2* variants are present in 30% (12/40). In accordance with earlier studies, none of the 3 African American/ Black patients in this group had *GJB2* variants [13].

In our cohort, *GJB2* variants were seen in 6 of 65 Hispanic Whites (9%). Earlier studies reported a range varying from 4 to 22% *GJB2* variants in Hispanic Whites. For instance, 22% biallelic *GJB2* variants were reported from a U.S. deaf population of 121 Hispanics by Pandya et al. [9, 21], while Sloan et al. reported a prevalence of 14% in their Hispanic cohort. However, Shan et al. [22] reported a much lower prevalence of *GJB2* mutations (less than 4%) in their study of Hispanic populations in the Bronx. These discrepancies in the prevalence of *GJB2* mutations among different studies could be due to differences in the study populations, sample sizes, and criteria used to define SNHL.

Causal variants in *MYO7A* were detected in 4 probands followed by *SLC26A4* and *STRC/CATSPER2* variants in 2 probands each. These three genes have been reported as relatively common causes of SNHL in previous studies from the U.S. and elsewhere [23–25]. As we observed in our study, *MYO7A* and *SLC26A4* are typically detected in patients with severe to profound SNHL, while *STRC/CATSPER2* variants cause mild to moderate HL. Additionally, as we observed, *STRC/CATSPER2* variants are predominantly copy number variants [26]. Interestingly, none of the African American/Black probands was found to have variants in these genes. Each of the other genes identified in this group involves only one proband, confirming the extreme heterogeneity of hereditary deafness. This was more prominent in Non-Hispanic Black/African Americans and Hispanic Whites, where the variants were rare and diverse. The heterogeneity in deafness gene variants were seen highest in the Hispanic Whites, which was also seen in a similar tertiary center [27]. It should be noted that the 3 Hispanic Blacks were negative for a causal gene variant. Some previous studies showed relatively more prevalent genes in certain populations. For instance, *MYO15A* variants were reported as one of the most frequently reported genetic causes of HL in North and Central Africa [28]. Similarly, *MYO15A* variants were found to be relatively common in the Middle East, Pakistan, Puerto Rico and a district of Brazil [29–31]. The scarcity of research on genetic HL in Africans impedes our comprehension of the distribution of causal HL genes. However, studies have shown that approximately 5% of African Americans carry variants in recurring genes, such as *OTOGL*, *COL11A2*, and *OTOF* [9, 32]. Our African American/Black cohort reported diverse geographical origins, including the Caribbean Islands, which may have contributed to the assortment of gene variants identified.

The diagnostic yield of our small Asian patient cohort was similar to that of a study by Sloan et al. [9] which evaluated 40 self-identified Asians and resulted in a diagnostic yield of 4%. This suggests that the low diagnostic yield may be due to the lack of high-quality studies with large numbers of Asian Americans. It is noteworthy that the only variant we detected in our Asian cohort, *SIX1* c.533G > C (p.Arg178Thr), was previously reported to cause HL in another Asian family [33].

Our study found that the phenotype related to the lowest diagnostic yield is unilateral HL. This is consistent with other studies showing that individuals with unilateral HL were less likely to receive a genetic diagnosis [34, 35]. Based on the evidence available, it can be assumed that genetic testing may not be the most appropriate first-line option in the diagnostic workup for individuals with unilateral HL. Instead, it may be more beneficial to focus on assessing potential environmental factors that may have contributed to HL. Therefore, before considering genetic testing, it is essential to evaluate the patient's medical history, environmental exposures, and any underlying medical conditions that may contribute to their HL.

We show that the SR for early-onset HL is higher compared to late-onset HL. This is consistent with previous research, that has shown that underlying genetic cause was more likely to be found in patients with congenital/prelingual HL, compared to those with post-lingual HL [35]. Furthermore, our study found no significant difference in SR between mild to moderate and severe to profound HL. This suggests that the severity of HL does not necessarily affect the likelihood of obtaining a diagnosis. Interestingly, our study found a difference in the diagnostic rate between syndromic and non-syndromic HL. The diagnostic rate for syndromic cases was 100%, while the diagnostic rate for non-syndromic cases was only 30%. This is likely due to the fact that environmental factors, such as CMV, can be more difficult to etiologically diagnose in non-syndromic HL [36]. Our study also found that the SR for a genetic HL diagnosis in cases with a positive family history was higher compared to cases with a negative family history. This is not surprising, as there is a greater chance of a genetic etiology for HL in multiplex families. Our study provides valuable insights into the factors that can affect the SR for HL etiology diagnosis. Figure 2 can be used as a guide to assess the diagnostic possibilities through genetic testing based on race and ethnicity as well as phenotypic variables of HL.

Some limitations of our study is that we obtained genetic test results from different laboratories instead of utilizing the same panel in all patients. This may have introduced a variance in diagnostic rates in different groups (Additional file 1: Table S2). Additionally, while our study provides valuable insights into the genetic causes of SNHL in racial and ethnic minorities of South Florida, it is essential to acknowledge the limitations associated with the small cohort size. The analysis was based on data from 136 patients presenting to the Hereditary Hearing Loss Clinic at the University of Miami, with a distribution of 115 (86%) White, 15 (11%) Black, and 6 (4%) Asian individuals, half of whom self-identified as Hispanic. The relatively small sample size, particularly in the Asian cohort, may not fully represent the diverse genetic landscape of the populations studied. Consequently, caution should be exercised in generalizing our findings to broader demographic groups. Larger and more comprehensive studies involving a more extensive and diverse patient population are warranted to enhance the reliability of our conclusions, particularly in addressing the genetic factors contributing to hearing loss in underrepresented populations.

## Conclusion

In conclusion, our study reveals that genetic testing for hearing loss in South Florida's Minority Population uncovers diverse DNA variants. We observe a greater variety of causative variants in racial and ethnic minorities compared to Non-Hispanic Whites. The identified variants in known hearing loss genes are less commonly found in racial/ethnic minorities, highlighting genetic heterogeneity. Our findings also indicate that the diagnostic rate of genetic studies varies by race in South Florida, with a 40% diagnostic rate for the genetic basis of hearing loss in this highly diverse population. Importantly, we note a persistently low diagnostic yield in some racial minorities, emphasizing the need to bridge the discovery gap in these groups. [22, 37].

## Methods

### Study population

A retrospective chart review was completed for 136 patients presenting for an etiological evaluation to the Hereditary HL clinic with a diagnosis of sensorineural hearing loss (SNHL) at the University of Miami Health System from 2017 to 2022. Patients were seen by an otologist, audiologist, and clinical geneticist during clinic encounters. Clinical evaluations included past medical and family histories and an otology exam as well as a thorough physical exam and eye exams. Investigations included a CT scan or MRI of the temporal bone, an EKG, and a kidney ultrasound. Patients with SNHL with or without additional findings were included. Patients with a clear environmental cause of HL were excluded. Only one affected person (proband) per family was included in the review.

Self-reported data on sex, family history, family origin, and phenotype were obtained from the patients` electronic medical records. Self-reported ethnicity included options of Hispanic and Non-Hispanic groups and race options were Asian, Black, White, American Indian/Alaska Native or Native Hawaiian/Pacific Islander.

### Phenotypic data

Clinical data collected from electronic medical records included audiological evaluations, pedigrees, HL age of onset, severity, and laterality. The onset of HL was categorized into two groups as early if the patient presented with HL before the age of 10, and late if the patient presented with HL at the ages above 11 years old. The most recent audiogram was used to group patients for unilateral vs bilateral HL categories. The severity was calculated from the pure tone average (PTA) hearing threshold between 500 and 4000 Hz (PTA_500-400_) in the most recent audiograms based on the better ear for bilateral HL. HL was defined based on PTA_500-400_ as follows: mild-moderate (20–70 dB) and severe-profound (> 71 dB) [38].

### Genetic data

Genetic testing was performed following pretest counseling by a certified genetic counselor or clinical geneticist. The utilized gene panels were from four different CLIA-certified laboratories and included 264 different genes. The range of genes included in each panel was 92–239 (Additional file 1: Table S2). Sequencing of coding regions and splice junctions and copy-number variant detection were performed. Confirmation of variants identified via next-generation sequencing was performed by the CLIA laboratories following their standard operational procedures and included Sanger sequencing, MLPA, and microarrays. Each variant reported by the laboratory was interpreted again by the study authors according to ACMG 2015 Guidelines and ClinGen HL Expert Panel (HL-EP) Specifications [39, 40]. A definitive genetic diagnosis was made based on the presence of pathogenic (P) or likely pathogenic (LP) variant(s) in a proband in accordance with the expected inheritance pattern. In AD or XL inheritance, if the proband is heterozygous or hemizygous for an LP or P variant, we considered this family solved. In AR inheritance, if the proband is homozygous or confirmed compound heterozygous by testing family members for LP/P variants, we considered this family solved. In mitochondrial inheritance, if the variant was LP/P in the proband, we considered this family solved. In AR inheritance, if the proband is heterozygous for two LP/P variants in the same gene but parental testing is not available, we considered this family possibly solved. If the proband has unique findings for a rare syndrome consistent with the gene-related phenotype, even if the variant is a variant of uncertain significance (VUS), we considered this family possibly solved.

### Supplementary Information

**Additional file 1: Table S1.** List of variants identified in families considered to be solved. **Table S2.** Hearing Loss Panels.

## Data Availability

All data generated or analyzed during this study are included in this published article and its supplementary information files.
