# Supplementary material for: Dispersed DNA variants underlie hearing loss in South Florida’s minority population
Source: Hum Genomics. 2023 Nov 24;17:103. doi: 10.1186/s40246-023-00556-7 (PMC10668374; doi:10.1186/s40246-023-00556-7)
Supplement: Supplementary file 1 — Additional file 1: Table S1. List of variants identified in families considered to be solved. Table S2. Hearing Loss Panels. [file 40246_2023_556_MOESM1_ESM.docx]

Supplemental Table 1

**List of variants identified in families considered to be solved**

| **Record ID** | **Family Hx** | **Ethnicity/**  **Race/**  **Family Origin** | **Gene** | **Variant** | **Zygosity** | **Population frequency^1^** | **PMID/**  **ClinVar ID** | **ACMG/HL-EP** **classification** | **Inheritance** | **Age of Onset** | **Phenotype** | **Other family members tested** | **Conclusion** |
| --- | --- | --- | --- | --- | --- | --- | --- | --- | --- | --- | --- | --- | --- |
| 76 | N | Non-Hispanic White Europe/  Italy | *ACTG1* | NM_001614.5  c.773C>T  p.Pro258Leu | Het | Absent | This Study | P | AD | Congenital (0-10yo) | Syndromic (Atypical Baraitser Winter Syndrome. with Retinitis pigmentosa) | N/A | S |
| 211 | N | Non-Hispanic White Eastern Europe/  Russia/  AJ decent | *AIFM1* | NM_004208.4  c.1412 G>A  p.Gly471Glu | Hem | Absent | This Study | VUS | X-linked | Post Lingual (11-30 yo) | Syndromic (AIFMI Related Disorder) | No | PS |
| 341 | N | Hispanic White Colombia | *ATP6V1B1* | NM_001692.4  c.1248+1G>C | Hom | Absent | PMID 9916796 | P | AR | Congenital (0-10yo) | Non Syndromic SNHL | No | S |
| 155 | N | Non-Hispanic White  Europe | *CDH23* | NM_022124.6  c.1515-12G>A | Comp  Het | 0.003% | This Study | VUS | AR | Congenital (0-10yo) | Non Syndromic SNHL | Yes (Mother and Father, confirmed to be in Trans) | PS |
|  |  |  |  | NM_022124.6  c.3598G>T  p.Asp1200Tyr |  | 0.0008% | This Study | VUS |  |  |  |  |  |
| 154 | N | Hispanic White Cuba | *COL11A1* | NM_001854.4  c.5009_5013delGTTGG  p.Ser1670Ilefs*2 | Het | Absent | This Study | P | AD | Congenital (0-10yo) | Syndromic SNHL (Stickler Syndrome) | No | S |
| 388 | Y | Non-Hispanic Black or African American Bahamas/ Haiti/ Dominican Republic | *COL11A1* | NM_001854.4  c.3816+1 G>A | Het | Absent | This Study | P | AD | Congenital (0-10yo) | Syndromic SNHL (Stickler Syndrome) | No | S |
| 382 | Y | Non-Hispanic African American USA | *COL2A1* | NM_001844.5  c.626G>A  p.Arg209Gln | Het | Absent | This Study | VUS | AD | Congenital (0-10yo) | Syndromic SNHL (Stickler Syndrome) | Yes (Mother and Father testing, confirmed in Trans) | PS |
|  |  |  |  | NM_001844.5  c.870+4_870+7del (Intronic) |  |  |  | VUS |  |  |  |  |  |
| 395 | Y | Hispanic White Venezuela/  Nicaragua | *COL2A1* | NM_001844.5  c.870+1G>A | Het | Absent | This Study | P | AD | Congenital (0-10yo) | Syndromic SNHL (Stickler Syndrome) | No | S |
| 160 | N | Non-Hispanic White Colombia/ White | *COL4A5* | NM_033380.3  c.4976+3A>G | Hem | Absent | This Study | VUS | X-linked | Congenital (0-10yo) | Syndromic SNHL (Alport Syndrome | Yes (Mother is carrier) | PS |
| 51 | N | Hispanic White Dominican Republic | *COL4A4* | NM_000092.5  c.2219dupC  p.Val741Cysfs*47 | Hom | Absent | This Study | P | AR | Congenital (0-10yo) | Syndromic SNHL (Alport Syndrome | No | S |
| 434 | Y | Hispanic white Puerto Rico | *CABP2* | NM_016366.3  c.590T>C  p.Ile197Thr | Hom | 0.05% | PMID: 26445815 | LP | AR | Congenital (0-10yo) | Non Syndromic SNHL | Yes (unaffectedbrother is Het for the variant) | S |
| 303 | Y | Non-Hispanic African American Haiti/  Denmark/  Portugal/ France | *EYA1* | NM_000503.6  c.966+5 G>T | Het | Absent | This Study | LP | AD | Congenital (0-10yo) | Syndromic SNHL (BOR Syndrome) | Yes (affected Parent is Het ) | S |
| 340 | Y | Hispanic White Cuba | *EYA1* | NM_000503.6  c.1653 T>A  p.Tyr551* | Het | Absent | This Study | LP | AD | Post Lingual (11-30) | Syndromic SNHL (BOR Syndrome) | No | S |
| 416 | N | Hispanic White Colombia | *GATA3* | DEL_Chr10:460,302-11,345,840  (pathogenic deletion of the entire coding sequence of GATA3) | Het | Absent | PMID: 20425828 | P | AD | Congenital (0-10yo) | Syndromic SNHL (Hypoparathyroidism, sensorineural deafness, and renal dysplasia) | No | S |
| 130 | Y | Hispanic White Brazil/ Cuba | *GJB2* | NM_004004.6  c.139 G>T  p.Glu47Ter | Comp Het | 0.03671% | PMID:  9336442 | P | AR | Congenital (0-10yo) | Non Syndromic SNHL | No | S |
|  |  |  |  | NM_004004.6  c.35delG  p.Gly12Valfs*2 |  | 0.9578% | PMID:  9285800 |  |  |  |  |  |  |
| 142 | N | Hispanic White Colombia/Jamaica/  China | *GJB2* | NM_004004.6  c.299_300delAT  p.His100Argfs*14 | Comp Het | 0.09023% | PMID: 20095872 | P | AR | Congenital (0-10yo) | Non Syndromic SNHL | Yes (Parents tested. Confirmed in Trans) | S |
|  |  |  |  | NM_004004.6  c.596 C>T  p. Ser199Phe |  | 0.01630% | ClinV:  VCV000189183.15 |  |  |  |  |  |  |
| 162 | N | Hispanic White Spain/  Cuba/  France | *GJB2* | NM_004004.6  c.35delG p.Gly12Valfs*2 | Hom | 0.9578% | PMID:  9285800 | P | AR | Congenital (0-10yo) | Non Syndromic SNHL | No | S |
| 183 | Y | Non-Hispanic White Ukraine | *GJB2* | NM_004004.6  c.35delG p.Gly12Valfs*2 | Hom | 0.9578% | PMID:  9285800 | P | AR | Congenital (0-10yo) | Non Syndromic SNHL | Yes (affected Sibling is Hom) | S |
| 189 | N | Non-Hispanic White Europe | *GJB2* | NM_004004.6  c.35delG p.Gly12fs*2 | Hom | 0.9578% | PMID:  9285800 | P | AR | Congenital (0-10yo) | Non Syndromic SNHL | No | S |
| 191 | U | Non-Hispanic White Southern Europe/ AJ | *GJB2* | NM_004004.6  c.35delG  p.Gly12Valfs*2 | Comp  Het | 0.9578% | PMID:  9285800 | P | AR | Congenital (0-10yo) | Non Syndromic SNHL | No | S |
|  |  |  |  | NM_006783.5  DEL_Chr13:20797176-21105944 |  | 0.01% | PMID: 11807148 |  |  |  |  |  |  |
| 216 | N | Hispanic White Cuba/  Colombia | *GJB2* | NM_004004.6  c.109 G>A  p.Val37Ile | Comp  Het | 8.345% | PMID: 12121355 | P | AR | Congenital (0-10yo) | Non Syndromic SNHL | No | S |
|  |  |  |  | NM_004004.6  c.35delG  p.Gly12Valfs*2 |  | 0.9578% | PMID:  9285800 |  |  |  |  |  |  |
| 265 | Y | Non-Hispanic White England/  Wales/  Scotland/  Ireland | *GJB2* | NM_004004.6  c.35delG p.Gly12Valfs*2 | Hom | 0.9578% | PMID:  9285800 | P | AR | Congenital (0-10yo) | Non Syndromic SNHL | No | S |
| 266 | N | Hispanic White Puerto Rico/Cuba | *GJB2* | NM_004004.6  c.35delG p.Gly12Valfs*2 | Hom | 0.9578% | PMID:  9285800 | P | AR | Congenital (0-10yo) | Non Syndromic SNHL | No | S |
| 317 | N | Non-Hispanic White Scotland/  Native American/American Eskimo/  Germany/  Ireland | *GJB2* | NM_004004.6  c.35delG p.Gly12Valfs*2 | Hom | 0.9578% | PMID:  9285800 | P | AR | Congenital (0-10yo) | Non Syndromic SNHL | No | S |
| 325 | N | Hispanic White Cuba | *GJB2* | NM_004004.6  c.35dupG  p.Val13Cfs*35 | Comp  Het | 0.01087% | PMID:  32645618 | P | AR | Congenital (0-10yo) | Non Syndromic SNHL | No | S |
|  |  |  |  | NM_004004.6  c.35delG  p.Gly12Valfs*2 |  | 0.9578% | PMID:  9285800 |  |  |  |  |  |  |
| 99 | Y | Non-Hispanic White White (specific origin unknown) | *GJB2* | NM_004004.6  c.269 T>C  p.Leu90Pro | Het | 0.119% | PMID: 10218527 | P | AR | Congenital (0-10yo) | Non Syndromic SNHL | No | S |
|  |  |  |  | NM_006783.4  309 kb deletion  GJB6-D43S1830 |  | Absent | PMID: 20301449 |  |  |  |  |  |  |
| 406 | N | Non-Hispanic White Poland/  Hungary/  Czech/ AJ | *LARS2* | NM_015340.4  c.180 G>C  p.Glu60Asp | Hom | 0.2412% | ClinV:  VCV000505259.4 | LP | AR | Congenital (0-10yo) | Syndromic SNHL (Perrault syndrome type 4) | No | S |
| 438 | Y | Non-Hispanic White Palestine | *LRP2* | NM_004525.3  c.11581 T>C  p. Cys3861Arg | Hom | Absent | This Study | LP | AR | Post Lingual (11-30) | Syndromic SNHL (Donnai Barrow syndrome) | No | S |
| 289 | Y | Non-Hispanic White  Ireland/  Netherlands | *SLC26A4* | NM_000441.2  c.85G>C  p.Glu29Gln | Het | 0.02289% | PMID: 11317356 | P | AR | Congenital (0-10yo) | Unknown ( SLC26A4 related disorder can be Pendred Syndrome or non-syndromic) | No | PS^+^ |
|  |  |  |  | NM_000441.2  c.1544+3_1544+6delGAGT |  | Absent | This Study | LP |  |  |  |  |  |
| 52 | N | Non-Hispanic Asian Vietnam/  India | *SIX1* | NM_005982.4  c.533G>C  p.Arg178Thr | Het | Absent | This Study | LP | AD | Post Lingual (11-30) | Syndromic SNHL (BOR Syndrome) | No | PS |
| 187 | Y | Hispanic White  Mexico/  Sweden | *STRC and CATSPER2* | Chr15q15.3 Deletion | Hom | Absent | PMID:  28574513 | P | AR | Congenital (0-10yo) | Non Syndromic SNHL | Yes (Affected sibling Hom) | S |
| 251 | N | Hispanic White Cuba | *STRC and CATSPER2* | Chr 15q15.3 Deletion | Hom | Absent | PMID:  28574513 | P | AR | Congenital (0-10yo) | Non Syndromic SNHL | No | S |
| 267 | Y | Non-Hispanic White Brazil | *WFS1* | NM_006005.3  c.409_424dupGGCCGTCGCGAGGCTG  p.Val142Glyfs*110 | Het | 0.01341% | PMID: 11161832 | P | AD | Congenital (0-10yo) | Non Syndromic SNHL | No | S |
| 227 | N | Hispanic White  Italy/  Greece/  Cuba/  Spain | *TBC1D24* | NM_001199107.2  c.724 C>T  p.Arg242Cys | Comp  Het | 0.006461% | PMID: 23806086 | P | AD/AR | Congenital (0-10yo) | Non Syndromic SNHL | Yes (Parents tested. Confirmed in Trans) | S |
|  |  |  |  | NM_001199107.2  c.641 G>A  p.Arg214His |  | 0.2242% | PMID: 22277662 | VUS |  |  |  |  |  |
| 297 | Y | Non-Hispanic Black/African American African American/Jamaica | *RTN4IP1* | NM_032730.5  c.308 G>A  p.Arg103His | Comp Het | 0.01002% | PMID: 26593267 | LP | AR | Congenital (0-10yo) | Syndromic SNHL (RTN4IP1-Related Disorder) | Yes (Parents tested. Confirmed in Trans) | PS |
|  |  |  |  | NM_032730.5  c.890 A>G  p. Tyr297Cys |  | 0.004006% | This Study | VUS |  |  |  |  |  |
| 169 | N | Hispanic White Mexico | *OTOG* | NM_001292063.2  c.6559 C>T  p.Arg2187* | Het | Absent | This Study | VUS | AR | Congenital (0-10yo) | Non Syndromic SNHL | No | PS^+^ |
|  |  |  |  | NM_001292063.2  c.8047+3 G>T |  |  |  |  |  |  |  |  |  |
| 396 | N | Non-Hispanic Black African American | *OTOGL* | NM_173591.3  c.2566_2569delAATT  p.Asn856Valfs*8 | Het | Absent | This Study | P | AR | Congenital (0-10yo) | Non Syndromic SNHL | No | PS^+^ |
|  |  |  |  | NM_173591.3  c.5992+5 G>A |  | 0.2% | PMID: 26969326 | LP |  |  |  |  |  |
| 288 | N | Non-Hispanic Black/ African American Dominica/ Haiti/  Bahamas | *OTOF* | NM_194248.3  c.2122C>T  p.Arg708* | Het | 0.002896% | PMID: 19636622 | P | AR | Congenital (0-10yo) | Non Syndromic SNHL | No | PS^+^ |
|  |  |  |  | NM_194248.3  c.1966delC  p.Arg656Glyfs*10 |  |  |  |  |  |  |  |  |  |
| 121 | N | Hispanic White Cuba/  Spain | *OTOF* | NM_194248.3  c.2485C>T  p.Gln829* | Comp  Het | 0.06427% | PMID: 12114484 | P | AR | Congenital (0-10yo) | Non Syndromic SNHL | Yes (Parents tested. Confirmed in Trans) | S |
|  |  |  |  | NM_194248.3  c.2348delGly  p.Gly783Alafs*17 |  | 0.01746% | PMID: 12525542 |  |  |  |  |  |  |
| 84 | N | Hispanic White Colombia | *MT-RNR1* | NC_012920  m.1555A>G | Homoplasmic | 0.1211% | PMID: 10577941 | P | Mito | Post Lingual (11-30) | Non Syndromic SNHL | No | S |
| 152 | N | Non-Hispanic White England/  Italy/  Ireland/  Scotland | *Mitochondrial genome* | NC_012920.1  m.8649_16084del17436 | Heteroplasmy 15% | Absent | PMID: 17157191 | P | Mito | Congenital (0-10yo) | Syndromic SNHL (Kearns-Sayre syndrome) | No | S |
| 209 | Y | Non-Hispanic White England  /Germany/  France | *MYO6* | NM_004999.4  c.2867 +1 G>A | Het | Absent | This Study | P | AD/AR | Post Lingual (11-30) | Non Syndromic SNHL | No | S |
| 237 | N | Non-Hispanic White Russia | *MYO7A* | NM_000260.4  c.5101 C>T  p.Arg1701* | Het | 0.01122% | PMID: 8900236 | P | AR | Congenital (0-10yo) | Syndromic SNHL (Usher Syndrome, Type 1) | No | PS^+^ |
|  |  |  |  | NM_000260.4  c.849+1 G>A |  | 0.000995% | PMID: 8900236 |  |  |  |  |  |  |
| 159 | Y | Hispanic white Honduras | *MYO7A* | NM_000260.3  c.73G>A  p.Gly25Arg | Hom | 0.002% | PMID: 9002678 | P | AR | Congenital (0-10yo) | Syndromic SNHL (Usher Syndrome, Type 1) | Yes (Parents tested. Confirmed in Trans) | S |
| 269 | Y | Hispanic White Peru | *MYO7A* | NM_000260.4  c.2283-1G>T | Comp  Het | Absent | This Study | LP | AR | Congenital (0-10yo) | Syndromic SNHL (Usher Syndrome, Type 1) | Yes (Parents tested. Confirmed in Trans) | S |
|  |  |  |  | NM_000260.4  c.4920delC  p.Glu1842fs*4 |  |  |  | LP |  |  |  |  |  |
| 394 | Y | Non-Hispanic White Germany/England | *MYO7A* | NM_000260.4  c.2164 G>C  p.Gly722Arg | Het | Absent | This Study | P | AD | Post Lingual (11-30) | Non Syndromic SNHL | No | S |
| 311 | Y | Hispanic White Puerto Rico | *MYO15A* | NM_016239.4  c.7226del  p.Pro2409Glnfs*8 | Hom | 0.02799% | PMID: 26226137 | P | AR | Congenital (0-10yo) | Non Syndromic SNHL | Yes (Affected sibling is Hom) | S |
| 423 | N | Non-Hispanic Black/African American African American/Haiti | *MYO15A* | NM_016239.4  c.8019delG  p.His2674Thrfs*64 | Comp  Het | Absent | This Study | P | AR | Congenital (0-10yo) | Non Syndromic SNHL | Yes (Parents tested. Confirmed in Trans) | S |
|  |  |  |  | NM_016239.4  c.8065delT  p.Trp2689Glyfs*49 |  | 0.01295% | ClinV:  VCV001197880.5 |  |  |  |  |  |  |
| 107 | Y | Hispanic White    Italy/ Spain/Mexico | *PCDH15* | NM_001384140.1  c.4211+1G>T | Comp Het | Absent | This Study | LP | AR | Congenital (0-10yo) | Non Syndromic SNHL | Yes (Parents tested. Confirmed in Trans) | S |
|  |  |  |  | NM_033056.3  c.3877C>T  p.Arg1293Trp |  | 0.0008% | This Study | LP |  |  |  |  |  |
| 430 | N | Non-Hispanic White Germany/Ukraine/Italy/Mexico | *SLC26A4* | NM_000441.2  c.165-1 G>A | Comp  Het | 0.03180% | PMID: 11375792 | P | AR | Congenital (0-10yo) | Syndromic SNHL (Pendred syndrome) | Yes (Parents tested. Confirmed in Trans) | S |
|  |  |  |  | NM_000441.2  c.707 T>C  p.Leu236Pro |  | 0.05964% | PMID: 10861298 |  |  |  |  |  |  |
| 168 | N | Hispanic White Cuba/Spain/Europe/AJ | *SUMF1* | NM_182760.4  c.463 T>C  p.Ser155Pro | Comp  Het | 0.1832% | PMID: 15146462 | P | AR | Congenital (0-10yo) | Syndromic SNHL (Multiple sulfatase deficiency) | Yes (Parents tested. Confirmed in Trans) | S |
|  |  |  |  | NM_182760.4  c.539 G>T  p.Trp180Leu |  | Absent | This Study | LP |  |  |  |  |  |
| 234 | N | Hispanic White Cuba | *TMC1* | NM_138691.3  c.236+1 G>A | Comp  Het | 0.005359% | PMID: 22607986 | P | AR | Congenital (0-10yo) | Non Syndromic SNHL | No | S |
|  |  |  |  | NM_138691.3  c.1939 T>C  p.Ser647Pro |  | 0.01629% | PMID: 21917145 | LP |  |  |  |  |  |
| 98 | N | Hispanic White Cuba | *TMPRSS3* | c.208delC  p.His70Thrfs*19 | Hom | 0.09649% | PMID: 21534946 | P | AR | Congenital (0-10yo) | Non Syndromic SNHL | No | S |
| 164 | N | Hispanic White Cuba/Argentina | *USH2A* | NM_206933.4  c.7475C>T  p.Ser2492Leu | Comp  Het | 0.01391% | PMID: 28041643 | LP | AR | Congenital (0-10yo) | Syndromic SNHL (Usher syndrome, type 2A) | Yes (Parents tested. Confirmed in Trans) | S |
|  |  |  |  | Deletion in Exon 70 |  | Absent | PMID: 23924366 | P |  |  |  |  |  |

VUS; Variant of Uncertain Significance, LB; Likely Benign, LP; Likely Pathogenic, P; Pathogenic, Hem; Hemizygous, Hom; Homozygous, Het; Heterozygous, Comp Het; Compound Heterozygous, Y; Yes, N; Negative; U; Unknown, S; Solved, PS; Potentially Solved, AJ; Ashkenazi Jewish; AD; Autosomal Dominant, AR; Autosomal Recessive, Mito; Mitochondrial

^1^ gnomAd population frequency is from the largest subpopulation

*The variants with ClinVar IDs have not been associated with hearing loss publications.

Patients with matching phenotype and appropriate segregation with parental testing were marked as solved

+If the patient had a matching phenotype but co-segregation was not examined (due to insurance, lack of availability, lost to follow up) they were marked as potentially solved.

**Supplemental Table 2**

Hearing Loss Panels

| ***GENEDX*** | ***BLUEPRINT*** | ***SEMA4*** | ***BMGDL*** |
| --- | --- | --- | --- |
| *ABHD12* | *ABHD12* | *ACTG1* | *ABHD12* |
| *ACTB* | *ACTG1* | *ADGRV1* | *ACTG1* |
| *ACTG1* | *ADCY1* | *AIFM1* | *ADCY1* |
| *ADCY1* | *ADGRV1* | *CACNA1D* | *AIFM1* |
| *AIFM1* | *AIFM1* | *CCDC50* | *ALMS1* |
| *ALMS1* | *ALMS1* | *CDH23* | *ATP6V181* |
| *ANKH* | *ANKH* | *CEACAM16* | *ATP6V182* |
| *ATP6V1B1* | *ARSG* | *CIB2* | *BCS1L* |
| *BDP1* | *ATP2B2* | *CLDN14* | *BSND* |
| *BSND* | *ATP6V1B1* | *CLRN1* | *CABP2* |
| *CABP2* | *ATP6V1B2* | *COCH* | *CACNA1D* |
| *CACNA1D* | *BCS1L* | *COL11A2* | *CATSPER2* |
| *CCDC50* | *BDP1* | *DFNA5* | *CCDC50* |
| *CD164* | *BSND* | *DFNB59* | *CD164* |
| *CDC14A* | *BTD* | *DIABLO* | *CDC14A* |
| *CDH23* | *C10ORF2* | *DIAPH1* | *CDH23* |
| *CEACAM16* | *CABP2* | *EDN3* | *CEACAM16* |
| *CHD7* | *CACNA1D* | *EDNRB* | *CEP78* |
| *CIB2* | *CATSPER2* | *ESPN* | *CHD7* |
| *CLDN14* | *CCDC50* | *ESRRB* | *CIB2* |
| *CLIC5* | *CD151* | *EYA1* | *CLDN14* |
| *CLPP* | *CD164* | *EYA4* | *CLIC5* |
| *CLRN1* | *CDC14A* | *GIPC3* | *CLPP* |
| *COCH* | *CDC42* | *GJB2* | *CLRN1* |
| *COL2A1* | *CDH23* | *GJB6* | *COCH* |
| *COL11A1* | *CDK9* | *GPSM2* | *COL11A1* |
| *COL11A2* | *CDKN1C* | *GRHL2* | *COL11A2* |
| *COL4A3* | *CEACAM16* | *GRXCR1* | *COL2A1* |
| *COL4A4* | *CEP250* | *HARS* | *COL4A3* |
| *COL4A5* | *CEP78* | *HGF* | *COL4A4* |
| *COL4A6* | *CHD7* | *ILDR1* | *COL4A5* |
| *CRYM* | *CHSY1* | *KARS* | *COL9A1* |
| *DCDC2* | *CIB2* | *KCNQ1* | *COL9A2* |
| *DFNA5* | *CLDN14* | *KCNQ4* | *CRYM* |
| *DIABLO* | *CLIC5* | *LHFPL5* | *DCDC2* |
| *DIAPH1* | *CLPP* | *LOXHD1* | *DFNA5 (GSDME)* |
| *DIAPH3* | *CLRN1* | *LRTOMT* | *DIABLO* |
| *DNMT1* | *COCH* | *MARVELD2* | *DIAPH1* |
| *DSPP* | *COL11A1* | *MITF* | *EDN3* |
| *EDN3* | *COL11A2* | *MSRB3* | *EDNRB* |
| *EDNRB* | *COL2A1* | *MT-RNR1* | *ELMOD3* |
| *ELMOD3* | *COL4A3* | *MYH14* | *EPS8* |
| *EPS8* | *COL4A4* | *MYH9* | *EPSBL2* |
| *ESPN* | *COL4A5* | *MYO15A* | *ESPN* |
| *ESRRB* | *COL4A6* | *MYO3A* | *ESRRB* |
| *EYA1* | *COL9A1* | *MYO6* | *EYA1* |
| *EYA4* | *COL9A2* | *MYO7A* | *EYA4* |
| *FAM65B* | *COL9A3* | *OPA1* | *FGF3* |
| *FGF3* | *CRYM* | *OTOA* | *FOXI1* |
| *FGFR1* | *DCAF17* | *OTOF* | *GATA3* |
| *FGFR2* | *DCDC2* | *OTOG* | *GIPC3* |
| *FGFR3* | *DFNA5* | *OTOGL* | *GJB1* |
| *FOXI1* | *DFNB31* | *P2RX2* | *GJB2* |
| *GATA3* | *DFNB59* | *PAX3* | *GJB3* |
| *GIPC3* | *DIABLO* | *PCDH15* | *GJB6* |
| *GJA1* | *DIAPH1* | *PEX1* | *GPR98(ADGRV1)* |
| *GJB2* | *DIAPH3* | *PEX10* | *GPSM2* |
| *GJB3* | *DLX5* | *PEX14* | *GRHL2* |
| *GJB6* | *DMXL2* | *PEX16* | *GRXCR1* |
| *GPR98* | *DNMT1* | *PEX19* | *GRXCR2* |
| *GPSM2* | *DSPP* | *PEX2* | *HARS* |
| *GRHL2* | *EDN3* | *PEX5* | *HARS2* |
| *GRXCR1* | *EDNRB* | *PEX6* | *HGF* |
| *HARS* | *EIF3F* | *PEX7* | *HSD17B4* |
| *HARS2* | *ELMOD3* | *PHYH* | *ILDR1* |
| *HGF* | *EPS8* | *POU3F4* | *KARS* |
| *HOMER2* | *EPS8L2* | *POU4F3* | *KCNE1* |
| *HSD17B4* | *ESPN* | *PRPS1* | *KCNJ10* |
| *ILDR1* | *ESRRB* | *PTPRQ* | *KCNQ1* |
| *KARS* | *EYA1* | *RDX* | *KCNQ4* |
| *KCNE1* | *EYA4* | *SERPINB6* | *KITLG* |
| *KCNJ10* | *FAM136A* | *SIX1* | *LARS2* |
| *KCNQ1* | *FAM65B* | *SIX5* | *LHFPL5* |
| *KCNQ4* | *FDXR* | *SLC26A4* | *LOXHD1* |
| *KITLG* | *FGF3* | *SMPX* | *LRTOMT* |
| *LARS2* | *FGFR3* | *SOX10* | *MARVELD2* |
| *LHFPL5* | *FITM2* | *STRC* | *MASP1* |
| *LRTOMT* | *FOXI1* | *TBC1D24* | *MITF* |
| *MARVELD2* | *GATA3* | *TECTA* | *MSRB3* |
| *MCM2* | *GIPC3* | *TIMM8A* | *MT-RNR1* |
| *MIR96* | *GJA1* | *TMC1* | *MYH14* |
| *MITF* | *GJB2* | *TMEM126A* | *MYH9* |
| *MSRB3* | *GJB3* | *TMIE* | *MYO15A* |
| *MT-CO1* | *GJB6* | *TMPRSS3* | *MYO3A* |
| *MT-RNR1* | *GPSM2* | *TPRN* | *MYO6* |
| *MT-TL1* | *GRHL2* | *TRIOBP* | *MYO7A* |
| *MT-TS1* | *GRXCR1* | *TSPEAR* | *NARS2* |
| *MYH14* | *GRXCR2* | *USH1C* | *NLRP3* |
| *MYH9* | *HARS* | *USH1G* | *OSBPL2* |
| *MYO15A* | *HARS2* | *USH2A* | *OTOA* |
| *MYO3A* | *HGF* | *WFS1* | *OTOF* |
| *MYO6* | *HOMER2* | *WHRN* | *OTOG* |
| *MYO7A* | *HOXB1* |  | *OTOGL* |
| *NDP* | *HSD17B4* |  | *P2RX2* |
| *NLRP3* | *ILDR1* |  | *PAX3* |
| *OPA1* | *KARS* |  | *PCDR15* |
| *OSBPL2* | *KCNE1* |  | *PDZD7* |
| *OTOA* | *KCNJ10* |  | *PJVK* |
| *OTOF* | *KCNQ1* |  | *PNPT1* |
| *OTOG* | *KCNQ4* |  | *POU3F4* |
| *OTOGL* | *KIT* |  | *POU4F3* |
| *P2RX2* | *LARS2* |  | *PRPS1* |
| *PAX3* | *LHFPL5* |  | *PTPN11* |
| *PCDH15* | *LMX1A* |  | *PTPRQ* |
| *PDZD7* | *LOXHD1* |  | *ROX* |
| *DFNB59* | *LRP2* |  | *RIPOR2* |
| *PMP22* | *LRTOMT* |  | *ROR1* |
| *PNPT1* | *MAN2B1* |  | *SERPINB6* |
| *POU3F4* | *MANBA* |  | *SIX1* |
| *POU4F3* | *MARVELD2* |  | *SIX5* |
| *POLR1D* | *MET* |  | *SLC26A4* |
| *PRPS1* | *MGP* |  | *SLC52A2* |
| *PTPRQ* | *MIR96* |  | *SMPX* |
| *RDX* | *MITF* |  | *SNAI2* |
| *S1PR2* | *MPZL2* |  | *SOX10* |
| *SALL1* | *MSRB3* |  | *STRC* |
| *SEMA3E* | *MT-ATP6* |  | *SYNE4* |
| *SERPINB6* | *MT-ATP8* |  | *TBC1D24* |
| *SIX1* | *MT-CO1* |  | *TECTA* |
| *SIX5* | *MT-CO2* |  | *TFAP2A* |
| *SLC17A8* | *MT-CO3* |  | *TIMM8A* |
| *SLC26A4* | *MT-CYB* |  | *TJP2* |
| *SLC26A5* | *MT-ND1* |  | *TMC1* |
| *SLC33A1* | *MT-ND2* |  | *TMIE* |
| *SLITRK6* | *MT-ND3* |  | *TMPRSS3* |
| *SMPX* | *MT-ND4* |  | *TNC* |
| *SNAI2* | *MTND4L* |  | *TPRN* |
| *SOX10* | *MT-ND5* |  | *TRIOBP* |
| *SOX2* | *MT-ND6* |  | *TSPEAR* |
| *STRC* | *MT-RNR1* |  | *TWNK* |
| *SYNE4* | *MT-RNR2* |  | *USH1C* |
| *TBC1D24* | *MT-TA* |  | *USH1G* |
| *TBX1* | *MT-TC* |  | *USH2A* |
| *TCOF1* | *MT-TD* |  | *WFS1* |
| *TECTA* | *MT-TE* |  | *WHRN* |
| *TIMM8A* | *MT-TF* |  |  |
| *TFAP2A* | *MT-TG* |  |  |
| *TJP2* | *MT-TH* |  |  |
| *TMC1* | *MT-TI* |  |  |
| *TMIE* | *MT-TK* |  |  |
| *TMPRSS3* | *MT-TL1* |  |  |
| *TNC* | *MT-TL2* |  |  |
| *TPRN* | *MT-TM* |  |  |
| *TRIOBP* | *MT-TN* |  |  |
| *TSPEAR* | *MT-TP* |  |  |
| *USH1C* | *MT-TQ* |  |  |
| *USH1G* | *MT-TR* |  |  |
| *USH2A* | *MT-TS1* |  |  |
| *WFS1* | *MT-TS2* |  |  |
| *WHRN* | *MT-TT* |  |  |
|  | *MT-TV* |  |  |
|  | *MT-TW* |  |  |
|  | *MT-TY* |  |  |
|  | *MYH14* |  |  |
|  | *MYH9* |  |  |
|  | *MYO15A* |  |  |
|  | *MYO3A* |  |  |
|  | *MYO6* |  |  |
|  | *MYO7A* |  |  |
|  | *NARS2* |  |  |
|  | *NDP* |  |  |
|  | *NLRP3* |  |  |
|  | *OSBPL2* |  |  |
|  | *OTOA* |  |  |
|  | *OTOF* |  |  |
|  | *OTOG* |  |  |
|  | *OTOGL* |  |  |
|  | *P2RX2* |  |  |
|  | *PAX3* |  |  |
|  | *PCDH15* |  |  |
|  | *PDE1C* |  |  |
|  | *PDZD7* |  |  |
|  | *PEX1* |  |  |
|  | *PEX26* |  |  |
|  | *PEX6* |  |  |
|  | *PISD* |  |  |
|  | *PNPT1* |  |  |
|  | *POLR1C* |  |  |
|  | *POLR1D* |  |  |
|  | *POU3F4* |  |  |
|  | *POU4F3* |  |  |
|  | *PRPS1* |  |  |
|  | *RDX* |  |  |
|  | *RMND1* |  |  |
|  | *RPS6KA3* |  |  |
|  | *S1PR2* |  |  |
|  | *SALL1* |  |  |
|  | *SALL4* |  |  |
|  | *SEMA3E* |  |  |
|  | *SERPINB6* |  |  |
|  | *SIX1* |  |  |
|  | *SIX5* |  |  |
|  | *SLC17A8* |  |  |
|  | *SLC19A2* |  |  |
|  | *SLC22A4* |  |  |
|  | *SLC26A4* |  |  |
|  | *SLC26A5* |  |  |
|  | *SLC29A3* |  |  |
|  | *SLC33A1* |  |  |
|  | *SLC52A2* |  |  |
|  | *SLC52A3* |  |  |
|  | *SLITRK6* |  |  |
|  | *SMAD4* |  |  |
|  | *SMPX* |  |  |
|  | *SNAI2* |  |  |
|  | *SOX10* |  |  |
|  | *SPATA5* |  |  |
|  | *STAG2* |  |  |
|  | *STRC* |  |  |
|  | *SUCLA2* |  |  |
|  | *SUCLG1* |  |  |
|  | *SYNE4* |  |  |
|  | *SYT2* |  |  |
|  | *TBC1D24* |  |  |
|  | *TBL1X* |  |  |
|  | *TCOF1* |  |  |
|  | *TECTA* |  |  |
|  | *TFAP2A* |  |  |
|  | *TIMM8A* |  |  |
|  | *TJP2* |  |  |
|  | *TMC1* |  |  |
|  | *TMEM132E* |  |  |
|  | *TMIE* |  |  |
|  | *TMPRSS3* |  |  |
|  | *TNC* |  |  |
|  | *TPRN* |  |  |
|  | *TRIOBP* |  |  |
|  | *TRMU* |  |  |
|  | *TSHZ1* |  |  |
|  | *TSPEAR* |  |  |
|  | *TUBB4B* |  |  |
|  | *TYR* |  |  |
|  | *USH1C* |  |  |
|  | *USH1G* |  |  |
|  | *USH2A* |  |  |
|  | *VCAN* |  |  |
|  | *WBP2* |  |  |
|  | *WFS1* |  |  |
|  | *XYLT2* |  |  |

| Panel Usage in Solved Cases: |
| --- |
| GENEDX 61% (33/54) |
| BLUEPRINT 13% (7/54) |
| SEMA4 4 % (2/54) |
| BMGDL 15% (8/54) |

*4 not included due to not being solved by HL panel. They were Solved by single gene test or

WES.

| Total Solve Rate of each Panel |
| --- |
| GENEDEX 42% (33/78) |
| BLUEPRINT 58% (7/12) |
| SEMA4 40% (2/5) |
| BMGDL 25% (8/32) |

* 9 cases not included due to no use of a HL panel. Single gene test or WES was used.

| Ethnic Distribution Per Gene Panel | | |
| --- | --- | --- |
|  | Hispanic (68) | Non Hispanic (68) |
| GENEDX | 45 (66%) | 33 (49%) |
| BLUEPRINT | 2 (3%) | 10 (15%) |
| SEMA4 | 4 (6%) | 1 (1%) |
| BMGDL | 12 (18%) | 21 (31%) |
|  |  |  |
|  | * 5 had other  miscellaneus  genetic testing done. | * 3 had other  miscellaneus  genetic testing done. |
